# Clear in California: Sesh+ nicotine pouches’ advertising under flavor restrictions

**DOI:** 10.18332/tpc/215182

**Published:** 2026-05-09

**Authors:** Joanne Chen Lyu, Artur Galimov, Ollie Ganz, Pamela M. Ling, Cassandra Stanton, Jenny E. Ozga

**Affiliations:** 1 TSET Health Promotion Research Center, Stephenson Cancer Center, University of Oklahoma Health Sciences Center, Oklahoma City, United States; 2 Department of Family and Preventive Medicine, College of Medicine, University of Oklahoma Health Sciences Center, Oklahoma City, United States; 3 Department of Population and Public Health Sciences, Keck School of Medicine, University of Southern California, Los Angeles, United States; 4 Rutgers Institute for Nicotine and Tobacco Studies, Rutgers Health, New Brunswick, United States; 5 Department of Health Behavior, Society, and Policy, Rutgers School of Public Health, Piscataway, United States; 6 Center for Tobacco Control Research and Education, University of California San Francisco, San Francisco, United States; 7 Behavioral Health and Health Policy Practice, Westat, Rockville, United States

**Keywords:** nicotine pouch, social media advertising, flavor ban, Clear in California

## Abstract

Sesh+ oral nicotine pouches (ONPs) are marketed as premium nicotine products and promoted regularly on social media platforms such as TikTok, Instagram, and X (formerly Twitter). In July 2025, Sesh+ ran 2623 ads on X; 561 (21.4%) explicitly promoted their ‘clear’ ONPs as being available for purchase in California using taglines like ‘Clear in California’, ‘California Clear’, and ‘Shop Sesh Clear in California’. California Senate Bill 793 (SB 793) prohibits retail sales of tobacco products, including ONPs that impart a ‘characterizing flavor’. Sesh+ markets its ‘clear’ ONPs on its website as ‘flavorless’, and the ‘clear’ pouches were present on California’s Unflavored Tobacco List in January 2026. The ‘Clear in California’ advertising raises concerns about geographically targeted marketing that turns regulatory compliance into a promotional message. By explicitly incorporating a state name and the term ‘clear’, the ads may imply special approval or regulatory endorsement, confusing consumers. Combined with youth-oriented social media content, this strategy may increase appeal to young audiences. Sesh+ advertising illustrates how ONP companies may transform legal compliance into a marketing asset, underscoring the need for continued surveillance of geographically targeted nicotine advertising and its implications for perceptions of harm, product appeal, and tobacco initiation.

## INTRODUCTION

Sesh+ oral nicotine pouches (ONPs), designed in Sweden and manufactured in the US, are marketed as premium quality. According to the brand website, they feature ‘pharmaceutical-grade’, ‘synthetic nicotine’, ‘gum-based’, ‘premium pouches’, and come in multiple strengths and flavors^1^. California Senate Bill 793 (SB 793, effective December 2022) prohibits retail sales of tobacco products, including ONPs, that impart a ‘characterizing flavor’^2^. SB 793 defines ‘characterizing flavor’ as ‘a distinguishable taste or aroma, or both, other than the taste or aroma of tobacco’ and further provides that ‘a tobacco product shall not be determined to have a characterizing flavor solely because of the use of additives or flavorings or the provision of ingredient information’^2^. Sesh+’s ‘clear’ ONPs are marketed on the brand’s website as ‘flavorless’^1^. If these products do not impart any ‘distinguishable taste or aroma’, their sale may be permissible in California as unflavored products, although compliance will ultimately require inclusion on the state’s forthcoming Unflavored Tobacco List^3^.

## COMMENTARY

**Figure 1:**
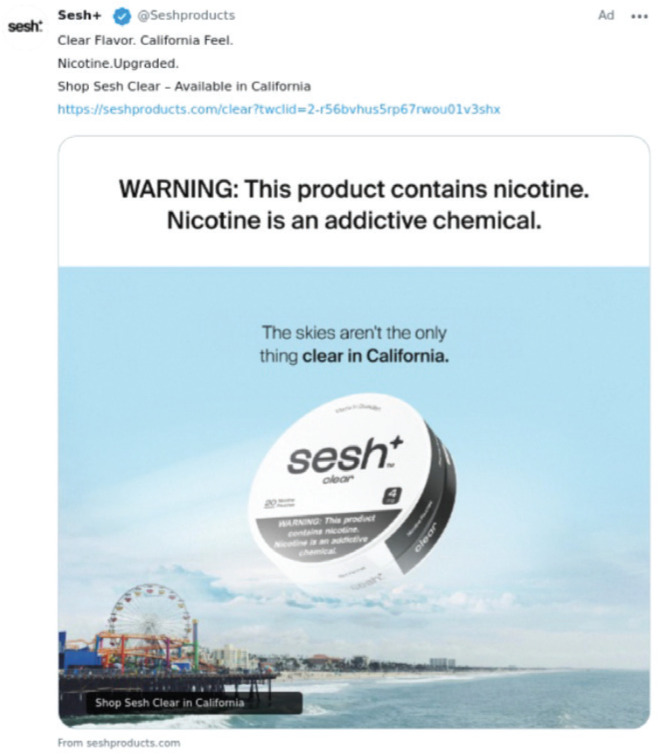
An example of Sesh+'s 'Clear in California' ads on X, first posted on 14 July 2025. In the ad, an oversized can of Sesh+ Clear nicotine pouches floats above the ocean with the Santa Monica Pier in the background. The tagline 'The skies aren’t the only thing clear in California' reinforces the connection between 'clear' and California’s iconic coastal imagery, alongside the call to action 'Shop Sesh Clear in California'

**Figure 2:**
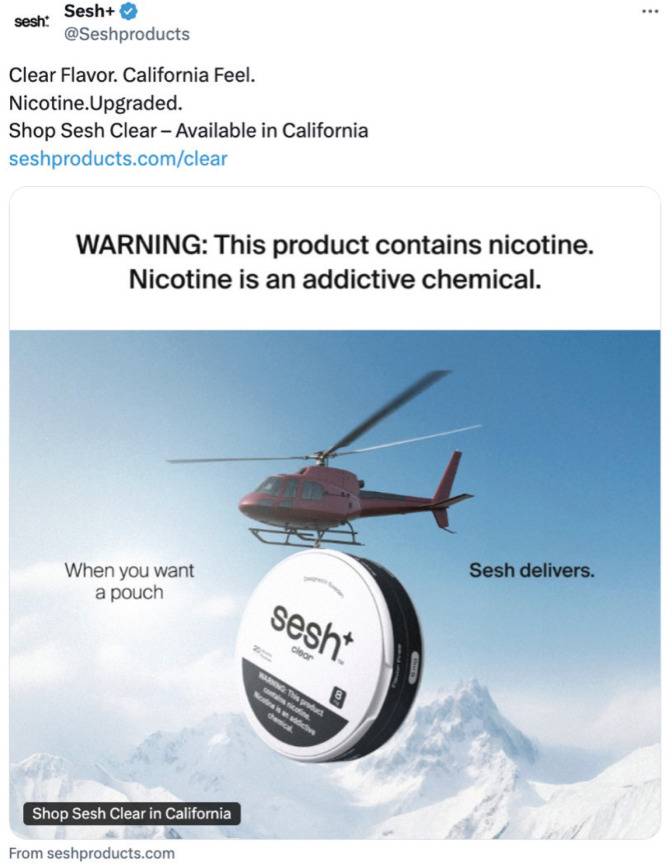
Another example of Sesh+'s 'Clear in California' ads on X, first posted on 4 July 2025. In the ad, a helicopter is shown airlifting an oversized can of Sesh+ Clear nicotine pouches over snowy mountains, with the tagline 'When you want a pouch, Sesh delivers'. The imagery positions the product as accessible anywhere, while pairing the 'Clear' name with the location-specific call to action 'Shop Sesh Clear in California'

Sesh+ markets their products regularly on social media platforms like TikTok and Instagram, and since July 2025, the brand has promoted its ‘clear’ ONPs on X (formerly Twitter) with taglines such as ‘Shop Sesh Clear in California’, ‘California Feel’, and ‘California Clear’ (Figures 1 and 2). Based on proprietary advertising intelligence data purchased from MediaRadar (July 2025), out of n=2623 Sesh+ ads on X in July 2025, 561 (21.4%) specifically promoted their ‘clear’ ONPs as being available for purchase in California. Although the ingredient list on the brand’s website does not include any characterizing flavors (e.g. mint, fruit, or candy flavors), it does list sweeteners such as maltitol and acesulfame potassium, which contribute to the product’s sweet taste^1^. Additionally, some ONPs labeled ‘clear’ are marketed as providing a distinct flavor experience. For example, Lucy’s ‘Clear’ pouches are described by some retailers as having a ‘tasty and cool wintergreen flavor’^4,5^, suggesting that the ‘clear’ descriptor may be ambiguous to consumers.

The Sesh+ website explicitly describes ‘clear’ nicotine pouches as a ‘flavorless option’1. While this product might be compliant with California’s flavor policy (SB 793), Sesh+’s ‘Clear in California’ advertising raises concerns. The tobacco industry historically has engaged in geographically concentrated marketing, such as disproportionately promoting menthol products in communities with higher proportions of Black residents and those with lower incomes^6,7^. Sesh+’s ‘Clear in California’ advertising represents a more explicit form of geographical targeting. In the context of SB 793, Sesh+ appears to strategically leverage the flavor prohibition by explicitly incorporating the state’s name and the term ‘clear’ (implying unflavored) into its tagline. ‘Clear in California’ may also create confusion, as it could be interpreted as suggesting endorsement, special approval, or having ‘cleared’ the policy in California. Such ambiguity has the potential to make the product more attractive to young people seeking special or novel items^8,9^. This potential appeal could be further reinforced by the brand’s social media marketing videos, which often feature youthful individuals and use humor and memes that may resonate with young audiences.

Although it is rare for widely disseminated ads to feature a state’s name, geographical references in local campaigns (e.g. ‘Newport Pleasure in New York’) have traditionally been used in cigarette marketing and somewhat recently by cigar brands. For instance, ‘Backwoods’ cigars features ‘California Range’ products on their website that are allegedly compliant with California’s flavor restrictions^10^. Sesh+’s ‘Clear in California’ represents an example of new nicotine products drawing from the traditional tobacco playbook. An analysis of Zyn, On!, and Velo ads revealed city-level targeting in Las Vegas, Charlotte, Atlanta, and Houston^11^. Another analysis of Velo, Zyn, and Black Buffalo ads found similar geographical targeting^12^. These examples suggest that regional marketing strategies are already being deployed in the growing ONP market, even without overt slogans like ‘Clear in California’. The impact of this concentrated marketing remains uncertain, but the use of legal compliance as a marketing theme, potentially extended to other geographical contexts, merits further scrutiny.

## CONCLUSION

The ‘Clear in California’ advertising leverages both flavor descriptors (‘clear’ implying unflavored) and regional identity to enhance product appeal. It exemplifies how some companies adapt to flavor restrictions, using implied legal compliance as a marketing hook. Further examination is needed to determine whether including jurisdiction names in advertising could mislead consumers about product safety and special approval, and whether ‘Clear in California’ may attract young people to use ONPs, potentially affecting the flavor restrictions’ goal of protecting youth. Ongoing surveillance and tracking of geographically targeted marketing strategies may also be necessary to assess their impact on consumer perceptions of harm, product appeal, and initiation, providing evidence to guide future regulatory approaches.

## Data Availability

Data sharing is not applicable to this article as no new data were created.
